# Supplementary figures and images for: Asymmetrical reliability of the Alda score favours a dichotomous representation of lithium responsiveness
Source: PLoS One. 2020 Jan 27;15(1):e0225353. doi: 10.1371/journal.pone.0225353 (PMC6984707; doi:10.1371/journal.pone.0225353)

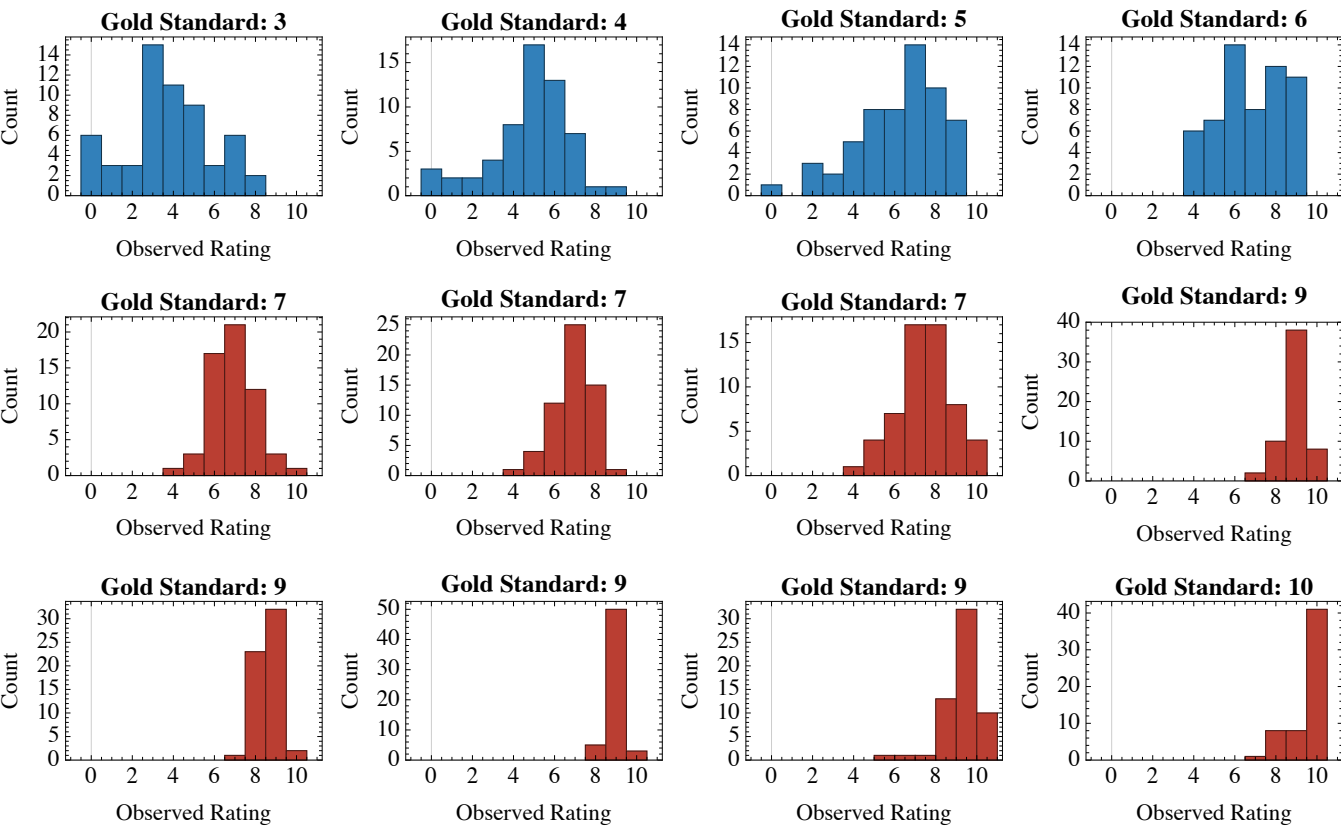

Supplement: S1 Fig — Histograms of ratings for each value of the ground truth Alda A-score. This figure was generated identically to Fig 2, but using the A-score data only. (PDF) [file pone.0225353.s001.pdf]
