# Supplementary material for: Asymmetrical reliability of the Alda score favours a dichotomous representation of lithium responsiveness
Source: PLoS One. 2020 Jan 27;15(1):e0225353. doi: 10.1371/journal.pone.0225353 (PMC6984707; doi:10.1371/journal.pone.0225353)
Supplement: S2 Fig — Mutual information between gold standard and observed Alda A-scores in relation to observation noise and the scale’s “raw” or dichotomized form. This figure was generated identically to Fig 3, but using the A-score data only. (PDF) [file pone.0225353.s002.pdf]

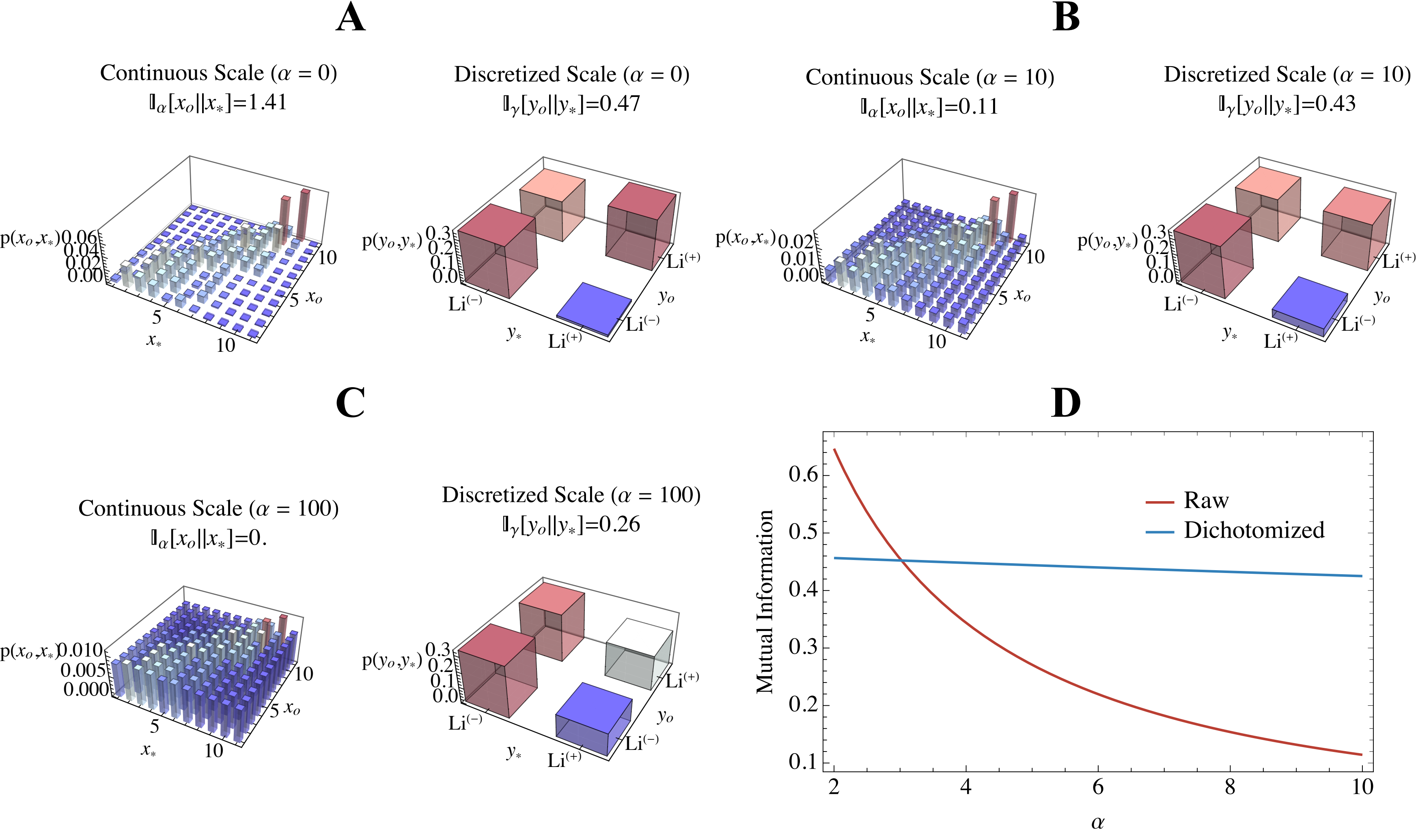

A
B
C
D
Continuous Scale (α = 0)
$\mathbb{I}_\alpha[x_o||x_*]$=1.41
Discretized Scale (α = 0)
$\mathbb{I}_\gamma[y_o||y_*]$=0.47
Continuous Scale (α = 10)
$\mathbb{I}_\alpha[x_o||x_*]$=0.11
Discretized Scale (α = 10)
$\mathbb{I}_\gamma[y_o||y_*]$=0.43
Continuous Scale (α = 100)
$\mathbb{I}_\alpha[x_o||x_*]$=0.
Discretized Scale (α = 100)
$\mathbb{I}_\gamma[y_o||y_*]$=0.26
$p(x_o,x_*)$
$p(y_o,y_*)$
x_*
x_o
y_*
y_o
$Li^{(-)}$
$Li^{(+)}$
Mutual Information
α
Raw
Dichotomized
