# Supplementary material for: Asymmetrical reliability of the Alda score favours a dichotomous representation of lithium responsiveness
Source: PLoS One. 2020 Jan 27;15(1):e0225353. doi: 10.1371/journal.pone.0225353 (PMC6984707; doi:10.1371/journal.pone.0225353)
Supplement: S6 File — PDF version of S3 File for those without Mathematica license. (PDF) [file pone.0225353.s008.pdf]

Code for Alda Scale Mutual Information Analysis in *Asymmetrical Reliability of the Alda Score favours a Dichotomous Representation of Lithium Response*

Abraham Nunes (nunes@dal.ca), Thomas Trappenberg, and Martin Alda
Dalhousie University, Halifax, Nova Scotia, Canada

Analysis of Reliability of the Total Alda Score

Table 1 Summarizing the Average Total Scores Across the Sites

```
wd = "path/to/directory/here/";
SetDirectory[wd];
```

In[4]:=
```
irrds = Import["S1_File.csv", "Dataset", HeaderLines→1];
```

In[5]:=

```
summary =Dataset[JoinAcross[Dataset[KeyValueMap[<|"Site"→#,"N Raters"→#2|>&,
    irrds[Counts, "Site"]]],
 Dataset[KeyValueMap[<|
 "Site"→#1,"Case 1"→#2[[1]], "Case 2"→#2[[2]],
 "Case 3"→#2[[3]],"Case 4"→#2[[4]],"Case 5"→#2[[5]],
 "Case 6"→#2[[6]],"Case 7"→#2[[7]],"Case 8"→#2[[8]],
 "Case 9"→#2[[9]],"Case 10"→#2[[10]],"Case 11"→#2[[11]],
 "Case 12"→#2[[12]]|>&,irrds[GroupBy["site"],
    Round[N@Mean[#],0.1]&, Table["Case "<>ToString[i], {i, 12}]]]]],
 "Site"]];

summary = TextGrid[Flatten[
    Catenate[{{{Normal@Keys[summary^T]}},{List@@@Normal@summary}}],1],
Frame→All]
```

Out[6]=

Site	N Raters	Case 1	Case 2	Case 3	Case 4	Case 5	Case 6	Case 7	Case 8	Case 9	Case 10	Case 11	Case 12
Consensus	1	8.	9.	6.	7.	9.	3.	5.	9.	3.	9.	5.	1.
Centre 1	9	8.4	8.6	6.6	6.9	9.2	3.	3.9	8.8	3.1	9.1	4.7	1.2
Centre 2	4	7.8	8.2	6.2	7.	8.8	3.2	4.	8.5	2.2	8.5	3.2	1.8
Centre 3	2	9.	8.5	6.5	5.5	9.	4.	7.5	9.	5.	8.	4.5	4.5
Centre 4	2	8.5	7.5	6.	5.	8.5	1.5	6.	9.	3.5	8.5	4.	1.5
Centre 5	4	8.	8.2	4.8	6.5	8.5	2.	3.	8.5	1.	8.2	4.5	1.5
Centre 6	2	7.5	7.5	4.	6.5	8.	1.5	3.	9.	0.	7.	3.	0.5
Centre 7	3	7.7	9.	4.3	7.	5.7	4.	1.3	9.	0.7	7.3	4.	2.
Centre 8	2	7.5	8.5	7.5	7.	9.	5.	7.5	8.5	3.5	8.5	6.	3.5
Centre 9	2	8.5	8.5	6.	7.	9.	3.	3.5	8.5	1.5	9.	4.	1.
Centre 10	2	9.5	9.	4.	6.	9.	1.	1.	9.	1.5	9.	4.	3.
Centre 11	1	7.	9.	4.	6.	9.	2.	3.	8.	0.	7.	0.	2.
Centre 12	1	8.	8.	5.	8.	9.	5.	6.	9.	4.	9.	8.	1.
Centre 13	1	7.	9.	4.	8.	9.	3.	6.	9.	3.	9.	6.	1.
Centre 14	7	8.	8.7	5.3	5.9	8.3	2.7	2.4	9.1	2.	8.3	4.4	1.1
Centre 15	6	8.	8.2	6.	8.	9.	4.2	3.	9.	4.2	8.8	3.7	0.3
Centre 16	3	8.	8.3	5.3	6.3	8.7	2.	4.	9.	4.3	8.	4.7	0.7
Centre 17	4	7.5	9.	5.5	6.5	5.	2.5	4.	7.2	4.8	8.8	1.2	2.
Centre 18	3	7.7	8.7	6.7	5.3	9.7	5.	6.	8.7	1.3	9.	3.7	0.3

Printed by Wolfram Mathematica Student Edition

Analysis of the Reliability under Noise

In[7]:=
```
X = Transpose[List@@@Normal@irrds[2;;, 4;;]];
y =  Flatten[List@@@Normal@irrds[{1}, 4;;]];
X = X[[Ordering[y]]];
y = y[[Ordering[y]]];
```

In[11]:=

```
RatingHistogram := Function[{X, y},
   Grid[
     ArrayReshape[{
       Table[Histogram[
           X[[i]],
           PlotLabel→ Style["Gold Standard: "<>ToString[y[[i]]], Black, Bold, 13
           ChartStyle→ColorData[63][[{ 1  y[[i]]≥7
                                       2  True    ]],
           PlotRange→{{-1, 11}, Automatic},
           Frame→True, FrameStyle→Directive[Black, 12],
           FrameLabel→{"Observed Rating", "Count"},
           PlotTheme→"Scientific"],
       {i, Length@y}]}, {3, 4}]]]
totalscorehistfig = RatingHistogram[X, y]
(*Export["total-score-hist-fig.pdf", totalscorehistfig, "AllowRasterization"→True];*)
```

Out[12]=

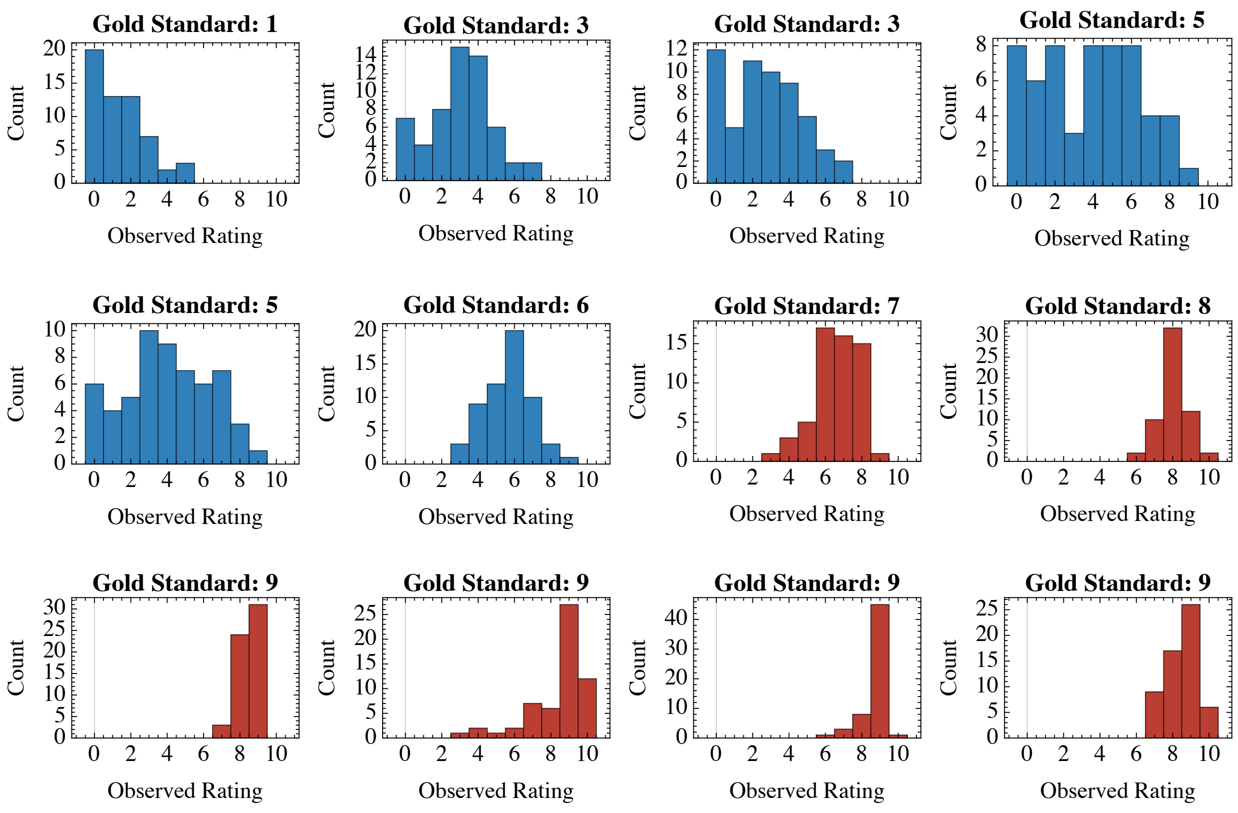

Estimation of Conditional Distributions

Let $n_i^{(k)} \in \mathbb{N}_+$ denote the number of raters who assigned Alda score $i \in \mathcal{A}$, with $\mathcal{A} = \{0, 1, ..., 10\}$, to an individual whose gold standard score is $k \in \mathcal{A}$. The vector of rating counts given gold standard

score k is $n^{(k)} = \left(n_i^{(k)}\right)_{i\in\mathcal{A}}$. The probability of $n^{(k)}$is multinomial with parameter vector $\theta^{(k)} = \left(\theta_i^{(k)}\right)_{i\in\mathcal{A}}$, which is itself Dirichlet distributed $\theta^{(k)} \approx \mathrm{Dir}(\theta \mid \alpha)$, where α are pseudocounts. The posterior of of $\theta^{(k)}$ given $n^{(k)}$ and α is Dirichlet with parameters $\alpha' = \left\{\alpha_i + n_i^{(k)} - 1\right\}_{i=0}^{10}$. The MAP estimate of $\theta^{(k)}$ given α and $n^{(k)}$ can then easily be shown to equal

$$\hat{\theta}_\alpha\left(n^{(k)}\right) = \left\{\frac{\alpha_i + n_i^{(k)} - 1}{\sum_{j=0}^{10}\left(\alpha_j + n_j^{(k)} - 1\right)}\right\}_{i=0}^{10}.$$

In the following analyses, we take $\hat{\theta}_\alpha\left(n^{(k)}\right)$ to be the conditional distribution over scores $\mathcal{A}$ for any given rater when the gold standard score is k.

The thresholded Alda scores are defined as $\mathcal{T} = \{\mathbb{1}_{i\geq 7} : \forall_{i\in\mathcal{A}}\}$, where $\mathbb{1}_x$ is an indicator function that evaluates to 1 if x is true, and 0 otherwise.

Letting $c_i^{(k)} = \mathbb{1}_{n_i^{(k)}\geq 7}$, $c_i^{(k)} \approx \mathrm{Multinomial}\left(\phi^{(k)}\right)$, and $\phi^{(k)} \approx \mathrm{Dir}(\phi \mid \xi)$, then we can estimate conditional distributions $\hat{\phi}_\xi\left(c^{(k)}\right)$ analogously to our process in the full score as follows:

$$\hat{\phi}_\xi\left(c^{(k)}\right) = \left\{\frac{\xi_i + c_i^{(k)} - 1}{\sum_{j=0}^{1}\left(\xi_j + c_j^{(k)} - 1\right)}\right\}_{i=0}^{1}.$$

Let x_* denote a “true” (gold standard) Alda score in $\mathcal{A}$ and x_o be a single observed rating on that same domain. Given uniform priors on the true classes, $\forall_{k\in\mathcal{A}}\ p(x_* = k) = \frac{1}{11}$, the joint distribution over true and observed (x_o) full-scale ratings is

$$p(x_o, x_*) = p(x_o \mid x_*)\, p(x_*) = \{p(x_o = i \mid x_* = k)\, p(x_* = k)\}_{i=0,1,\ldots,10}^{k=0,1,\ldots,10} = \left\{\frac{1}{11}\,\hat{\theta}_\alpha\left(n^{(k)}\right)\right\}_{k=0,1,\ldots,10}.$$

Similarly for the binarized classes, we have a prior of $p(y_*) = \left\{\frac{7}{11}, \frac{4}{11}\right\}$, and the joint distribution is thus

$$p(y_o, y_*) = p(y_o \mid y_*)\, p(y_*) = \{p(y_o = i \mid y_* = k)\, p(y_* = k)\}_{i=0,1}^{k=0,1} = \left\{\frac{7}{11}\,\hat{\phi}_\xi\left(c^{(0)}\right), \frac{4}{11}\,\hat{\phi}_\xi\left(c^{(1)}\right)\right\}.$$

In[13]:=
```
DirMulEstimate[α_]:= Function[Q,
    Module[{λ},
    λ = Flatten@Table[
        α[[i]] + { 0                                                              Flatten[Positi
                 { Tally[Q][[Flatten[Position[Tally[Q][[All, 1]], i]], 2]]  True
        {i,Length@α}];
    λ/Total[λ]
]]
```

Compute the conditional distribution:

In[14]:=
```
Q[α_:2] := {
    DirMulEstimate[Table[α, 11]][Table[Min[{Max[{X[[1]][[i]]-1, 0}], 10}], {i, Length@X[[
    DirMulEstimate[Table[α, 11]][X[[1]]],
    Mean[Table[DirMulEstimate[Table[α, 11]][X[[k]]], {k, 1, 3}]],
    Mean[Table[DirMulEstimate[Table[α, 11]][X[[k]]], {k, 2, 3}]],
    Mean[Table[DirMulEstimate[Table[α, 11]][X[[k]]], {k, 2, 5}]],
    Mean[Table[DirMulEstimate[Table[α, 11]][X[[k]]], {k, 4, 5}]],
    DirMulEstimate[Table[α, 11]][X[[6]]],
    DirMulEstimate[Table[α, 11]][X[[7]]],
    DirMulEstimate[Table[α, 11]][X[[8]]],
    Mean[Table[DirMulEstimate[Table[α, 11]][X[[k]]], {k, 9, 12}]],
    Mean[Table[DirMulEstimate[Table[α, 11]][X[[k]]+1], {k, 9, 12}]]
}ᵀ
```

Thresholded Alda scores and joint distribution function:

In[15]:=
```
ThresholdAlda[X_]:=Table[{ 0  x<7
                           1  True , {x, X}];
J[α_:2]:= {
 7/11 DirMulEstimate[Table[α, 2]][ThresholdAlda@Flatten@X[[;;6]]+1],
 4/11 DirMulEstimate[Table[α, 2]][ThresholdAlda@Flatten@X[[7;;]]+1]
}
```

Joint Distribution over True and Observed Scores

In[17]:=
```
jointdistribaldascorefig = ArrayPlot[(Q[2]×Table[1/11, {i, 11}, {i, 11}]),
PlotLabel→Style["Joint Distribution over True and Observed Alda Score", Black, 13],
PlotTheme→"Scientific",
ColorFunction→"ThermometerColors",
PlotLegends→Automatic,
Frame→True,FrameStyle→Directive[Black, 13],
FrameLabel→{"Observed Alda Score", "True Alda Score"},
 FrameTicks→{{Range[11], Range[0, 10]}ᵀ, {Range[11], Range[0, 10]}ᵀ}, ImageSize→350]

(*Export["jointdistributionaldascore.png", jointdistribaldascorefig, ImageResolution→500,
```

Out[17]=

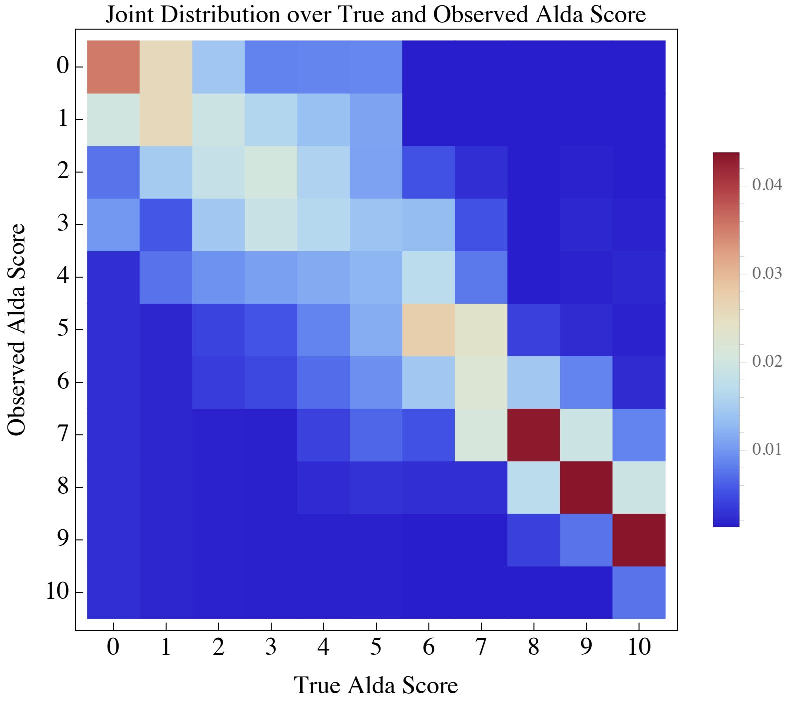

We can compute the mutual information between the observed and gold standard Alda scores (continuous and thresholded) as follows:

$$\mathbb{I}_{\alpha}[x_o \,||\, x_*] = \sum_{i\in\mathcal{A}} \sum_{j\in\mathcal{A}} p(x_o = i,\, x_* = j)\, \mathrm{Log}\left[\frac{p(x_o=i, x_*=j)}{p(x_o=i)\, p(x_*=j)}\right],$$

and

$$\mathbb{I}_{\xi}[y_o \,||\, y_*] = \sum_{i\in\mathcal{T}} \sum_{j\in\mathcal{T}} p(y_o = i,\, y_* = j)\, \mathrm{Log}\left[\frac{p(y_o=i, y_*=j)}{p(y_o=i)\, p(y_*=j)}\right],$$

respectively. We maintain the hyperparameters α and ξ in the notation since they control the level of

uncertainty in the conditional distributions. Higher values of α and ξ yield conditional distributions $\hat{\theta}_\alpha(n^{(k)})$ and $\hat{\phi}_\xi(c^{(k)})$ with higher uncertainty (i.e they are noisier).

In[18]:=
```
Pxy[α_]:= (Q[α]×Table[1/11, {i, 11}, {i, 11}]);
Px[α_]:= (Total@Pxy[α]);
Py[α_]:= Total[Pxy[α]ᵀ];
Jxy[α_]:= J[α];
Jx[α_]:= (Total@Jxy[α]);
Jy[α_]:= Total[Jxy[α]ᵀ];
```

Letting $\xi = \frac{11}{2}\alpha$ in order to provide some equivalence between the priors, we can plot $\mathbb{I}_\alpha[x_o \,||\, x_*]$ and $\mathbb{I}_\xi[y_o \,||\, y_*]$ across increasing levels of uncertainty.

In[24]:=
```
AldaMI[α_]:=N@Total[Total[Pxy[α] Log[Pxy[α]/(Px[α]⊗Py[α])]]]
DiscreteAldaMI[α_]:=N@Total[Total[Jxy[α] Log[Jxy[α]/(Jx[α]⊗Jy[α])]]]
```

In[26]:=

```
mitotalaldafig = Plot[
    {AldaMI[α], DiscreteAldaMI[α]}, {α, 2, 10},
    PlotStyle→63, Frame→True, FrameStyle→Directive[Black,15],
    FrameLabel→{"α", "Mutual Information"},
    PlotTheme→"Scientific",
    PlotLegends→Placed[{
        Style["Raw", Black, 16],
        Style["Dichotomized", Black, 16]},
        {0.75, 0.75}],
    ImageSize→350]
(*Export["~/Desktop/mutualinformationaldascores.png",
        mitotalaldafig,
        ImageResolution→500,
        "AllowRasterization"→True];*)
```

Out[26]=

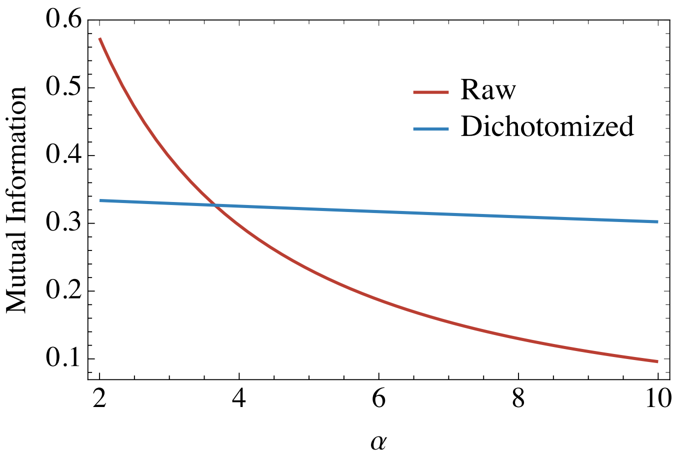

We therefore note a whole set of conditions after approximately $\alpha = 3.5$ in which discretization is more informative than the continuous distribution.

But why is this the case? The plots below show that asymmetry may be the culprit.

In[27]:=

```
partA = GraphicsGrid[{{DiscretePlot3D[Pxy[0.0001][[i,j]],{i,1,11}, {j,1,11}, ExtentSize→1
 PlotLabel→Style["Continuous Scale (α = 0)\n𝕀α[xo||x*]="<>ToString[Round[Re@AldaMI[0.0001]
 PlotTheme→"Scientific", ColorFunction→"ThermometerColors", AxesLabel→{"x*", "xo", "p(xo,x
 AxesStyle→Directive[Black, 13]],
 DiscretePlot3D[Jxy[ 11/2 ×0.0001][[i,j]],{i,1,2}, {j,1,2}, ExtentSize→1/2,
 PlotLabel→Style["Discretized Scale (α = 0)\n𝕀γ[yo||y*]="<>ToString[Round[DiscreteAldaMI[0
 PlotTheme→"Scientific", ColorFunction→"ThermometerColors", AxesLabel→{"y*", "yo", "p(yo,y
 AxesStyle→Directive[Black, 13],
 Ticks→{{{1, 2}, {"Li(-)", "Li(+)"}}ᵀ, {{1, 2}, {"Li(-)", "Li(+)"}}ᵀ, Automatic}]}}, ImageSize
```

```
partB = GraphicsGrid[{{DiscretePlot3D[Pxy[10][[i,j]],{i,1,11}, {j,1,11}, ExtentSize→1/2,
 PlotLabel→Style["Continuous Scale (α = 10)\n𝕀α[xo||x*]="<>ToString[Round[Re@AldaMI[10], 0
 PlotTheme→"Scientific", ColorFunction→"ThermometerColors", AxesLabel→{"x*", "xo", "p(xo,x
 AxesStyle→Directive[Black, 13]],
 DiscretePlot3D[Jxy[ 11/2×10][[i,j]],{i,1,2}, {j,1,2}, ExtentSize→1/2,
 PlotLabel→Style["Discretized Scale (α = 10)\n𝕀γ[yo||y*]="<>ToString[Round[DiscreteAldaMI[
 PlotTheme→"Scientific", ColorFunction→"ThermometerColors", AxesLabel→{"y*", "yo", "p(yo,y
 AxesStyle→Directive[Black, 13],
 Ticks→{{{1, 2}, {"Li(-)", "Li(+)"}}ᵀ, {{1, 2}, {"Li(-)", "Li(+)"}}ᵀ, Automatic}]}}, ImageSize

partC = GraphicsGrid[{{DiscretePlot3D[Pxy[100][[i,j]],{i,1,11}, {j,1,11}, ExtentSize→1/2,
 PlotLabel→Style["Continuous Scale (α = 100)\n𝕀α[xo||x*]="<>ToString[Round[Re@AldaMI[100],
 PlotTheme→"Scientific", ColorFunction→"ThermometerColors", AxesLabel→{"x*", "xo", "p(xo,x
 AxesStyle→Directive[Black, 13]],
 DiscretePlot3D[Jxy[ 11/2×100][[i,j]],{i,1,2}, {j,1,2}, ExtentSize→1/2,
 PlotLabel→Style["Discretized Scale (α = 100)\n𝕀γ[yo||y*]="<>ToString[Round[DiscreteAldaMI
 PlotTheme→"Scientific", ColorFunction→"ThermometerColors", AxesLabel→{"y*", "yo", "p(yo,y
 AxesStyle→Directive[Black, 13],
 Ticks→{{{1, 2}, {"Li(-)", "Li(+)"}}ᵀ, {{1, 2}, {"Li(-)", "Li(+)"}}ᵀ, Automatic}]}}, ImageSize

gridlab[x_] := Style[x, Black, Bold, 28, FontFamily→"Times New Roman"]
(*totalscoreasymmetrygrid = Grid[{
    gridlab[#]&/@{"A", "B"},
    {partA, partB},
    gridlab[#]&/@{"C", "D"},
    {partC, mitotalaldafig}}];
Export["totalscoreasymmetrygrid.pdf", totalscoreasymmetrygrid, "AllowRasterization"→True]

partA
partB
partC
mitotalaldafig
```

Out[31]=

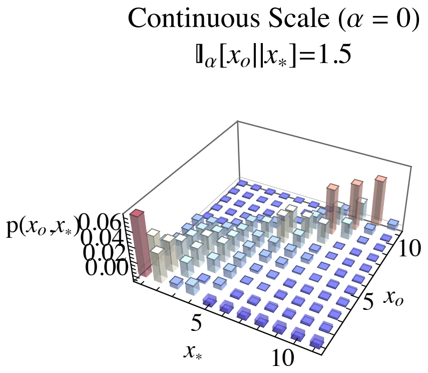

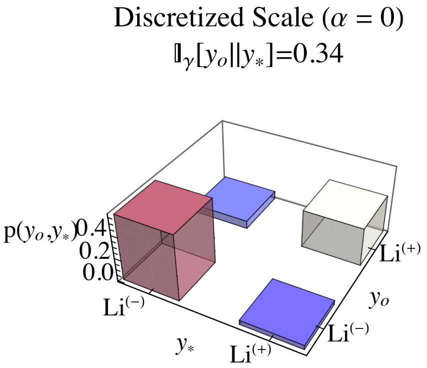

Out[32]=

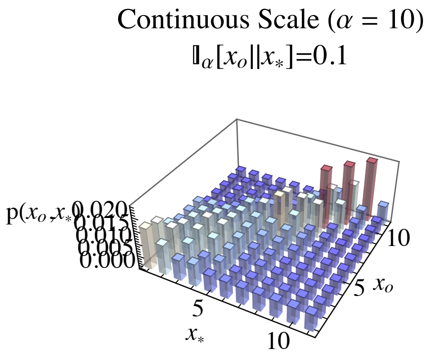

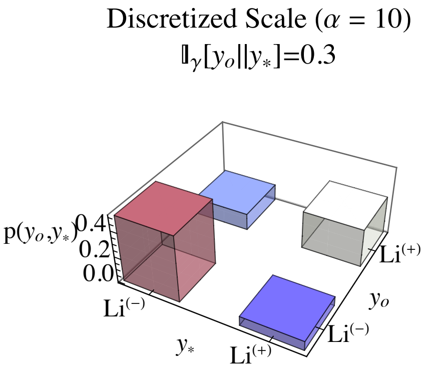

Out[33]=

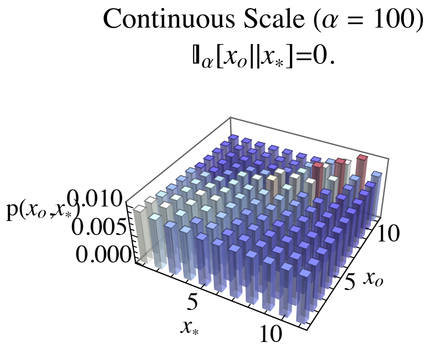

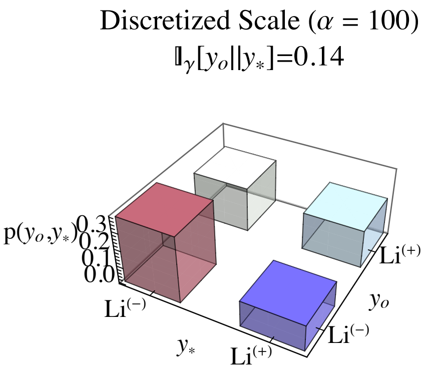

Printed by Wolfram Mathematica Student Edition

Out[34]=

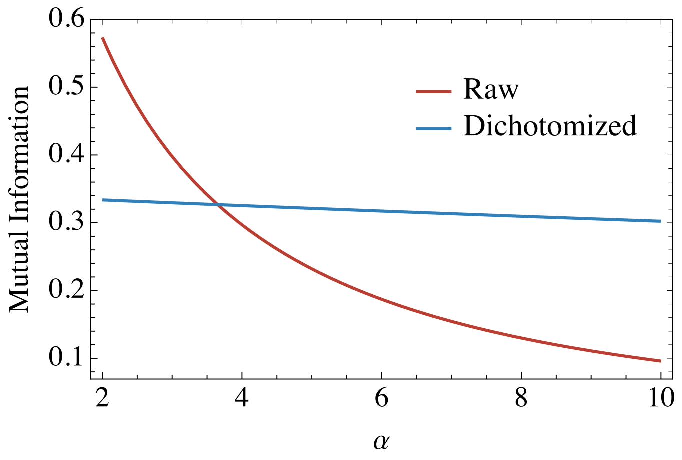

Supplemental Analysis: Mutual Information of the Alda A-Score

Here we repeat the analyses done for the total score using only the Alda A-scores.

Summary Table

In[35]:=
```
irrdsA = Import["S2_File.csv", "Dataset", HeaderLines→1];
```

In[36]:=
```
summaryA =Dataset[JoinAcross[Dataset[KeyValueMap[<|"Site"→#,"N Raters"→#2|>&,irrdsA[Count
 Dataset[KeyValueMap[<|
  "Site"→#1,"Case 1"→#2[[1]], "Case 2"→#2[[2]],
  "Case 3"→#2[[3]],"Case 4"→#2[[4]],"Case 5"→#2[[5]],
  "Case 6"→#2[[6]],"Case 7"→#2[[7]],"Case 8"→#2[[8]],
  "Case 9"→#2[[9]],"Case 10"→#2[[10]],"Case 11"→#2[[11]],
  "Case 12"→#2[[12]]|>&,irrdsA[GroupBy["site"],Round[N@Mean[#],0.1]&, Table["Case "<>ToStr
 "Site"]];
summaryA = TextGrid[Flatten[
    Catenate[{{{Normal@Keys[summaryAᵀ]}},{List@@@Normal@summaryA}}],1],
Frame→All]
```

Out[37]=

Site	N Raters	Case 1	Case 2	Case 3	Case 4	Case 5	Case 6	Case 7	Case 8	Case 9	Case 10	Case 11	Case 12
Consensus	1	9.	9.	7.	7.	10.	4.	7.	9.	5.	9.	6.	3.
Centre 1	9	9.1	8.6	7.3	7.1	9.9	4.4	6.9	9.	6.6	9.4	6.9	3.2
Centre 2	4	8.8	8.8	8.	7.2	9.5	5.2	7.8	8.5	5.8	8.8	6.	4.8
Centre 3	2	10.	9.5	6.5	6.5	10.	6.	8.5	9.	8.	8.	6.	7.5
Centre 4	2	9.	8.	6.	5.	9.	2.5	8.	9.	6.5	8.5	7.	2.5
Centre 5	4	9.	8.5	6.	6.5	9.5	3.	6.2	8.8	3.2	8.5	7.	3.
Centre 6	2	8.5	7.5	5.5	6.5	9.	3.5	7.	9.	3.5	8.	6.5	2.
Centre 7	3	9.	9.	6.3	7.	10.	6.	8.3	9.	6.7	8.3	8.	6.3
Centre 8	2	9.	8.5	8.	7.	10.	7.	9.5	10.	9.	5.5	8.5	7.5
Centre 9	2	9.5	8.5	8.	7.	10.	4.5	8.5	9.	7.	9.5	7.	3.
Centre 10	2	10.	9.	6.	6.	10.	2.5	7.	9.	6.5	9.	8.	7.
Centre 11	1	8.	9.	6.	7.	10.	4.	6.	8.	4.	8.	4.	4.
Centre 12	1	9.	8.	6.	8.	10.	7.	10.	9.	7.	9.	9.	3.
Centre 13	1	9.	9.	6.	8.	10.	5.	8.	9.	6.	9.	9.	3.
Centre 14	7	9.	8.9	7.	6.9	9.7	4.3	6.9	9.1	5.7	8.9	7.4	3.6
Centre 15	6	8.	8.2	7.	8.	9.	6.3	7.8	9.	7.2	9.	6.2	1.7
Centre 16	3	9.	8.3	7.3	6.7	10.	5.	8.3	9.	7.7	9.	7.	2.3
Centre 17	4	8.5	9.	6.8	7.	7.8	5.	6.2	9.	7.	9.5	5.	4.8
Centre 18	3	8.7	8.7	7.3	5.7	9.7	5.7	8.	8.7	4.7	9.	6.	3.3

In[38]:=
```
X = Transpose[List@@@Normal@irrdsA[2;;, 4;;]];
y =  Flatten[List@@@Normal@irrdsA[{1}, 4;;]];
X = X[[Ordering[y]]];
y = y[[Ordering[y]]];
```

Plot the histograms

In[42]:=
```
ascorehistfig = RatingHistogram[X, y]
(*Export["a-score-hist-fig.pdf", ascorehistfig, "AllowRasterization"→True];*)
```

Out[42]=

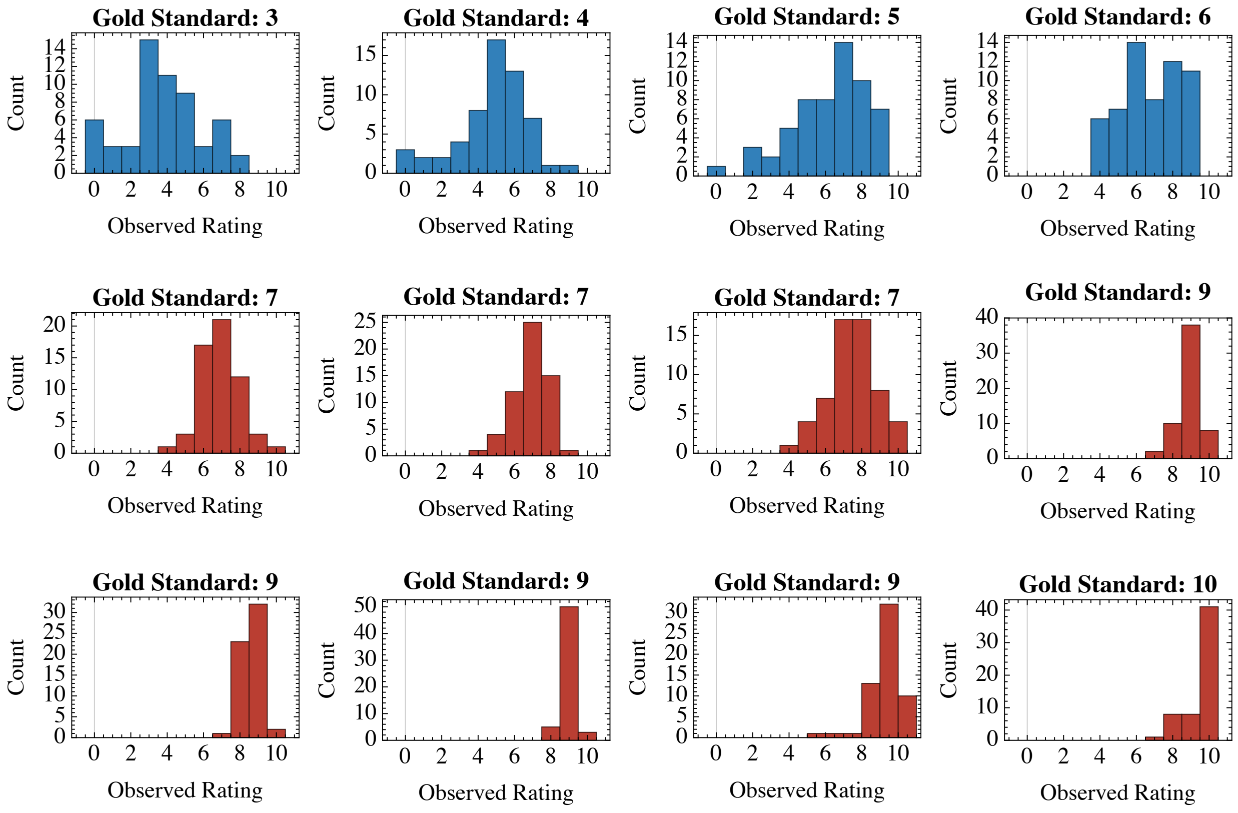

In[43]:=
```
Q[α_:2] := {
    DirMulEstimate[Table[α, 11]][Table[Min[{Max[{X[[1]][[i]]-1, 0}], 10}], {i, Length@X[[
    DirMulEstimate[Table[α, 11]][X[[1]]],
    DirMulEstimate[Table[α, 11]][X[[1]]],
    DirMulEstimate[Table[α, 11]][X[[1]]],
    DirMulEstimate[Table[α, 11]][X[[2]]],
    DirMulEstimate[Table[α, 11]][X[[3]]],
    DirMulEstimate[Table[α, 11]][X[[4]]],
    Mean[Table[DirMulEstimate[Table[α, 11]][X[[k]]], {k, 5, 7}]],
    (Mean[Table[DirMulEstimate[Table[α, 11]][X[[k]]], {k, 5, 7}]] +
       Mean[Table[DirMulEstimate[Table[α, 11]][X[[k]]], {k, 8, 11}]])/2,
    Mean[Table[DirMulEstimate[Table[α, 11]][X[[k]]], {k, 8, 11}]],
    DirMulEstimate[Table[α, 11]][X[[12]]]
}ᵀ
```

Thresholded Alda scores and joint distribution function:

In[44]:=
```
ThresholdAlda[X_]:=Table[{ 0  x<7
                           1  True , {x, X}];
J[α_:2]:= {
 7/11 DirMulEstimate[Table[α, 2]][ThresholdAlda@Flatten@X[[;;6]]+1],
 4/11 DirMulEstimate[Table[α, 2]][ThresholdAlda@Flatten@X[[7;;]]+1]
}
```

Joint Distribution over True and Observed Scores

In[46]:=

```
jointdistribaldaAscorefig = ArrayPlot[(Q[2]×Table[1/11, {i, 11}, {i, 11}]),
PlotLabel→Style["Joint Distribution over True and Observed Alda-A Score", Black, 13],
PlotTheme→"Scientific",
ColorFunction→"ThermometerColors",
PlotLegends→Automatic,
Frame→True,FrameStyle→Directive[Black, 13],
FrameLabel→{"Observed Alda-A Score", "True Alda-A Score"},
 FrameTicks→{{Range[11], Range[0, 10]}ᵀ, {Range[11], Range[0, 10]}ᵀ}, ImageSize→350]

(*Export["jointdistributionaldascore.png", jointdistribaldaAscorefig, ImageResolution→500
```

Out[46]=

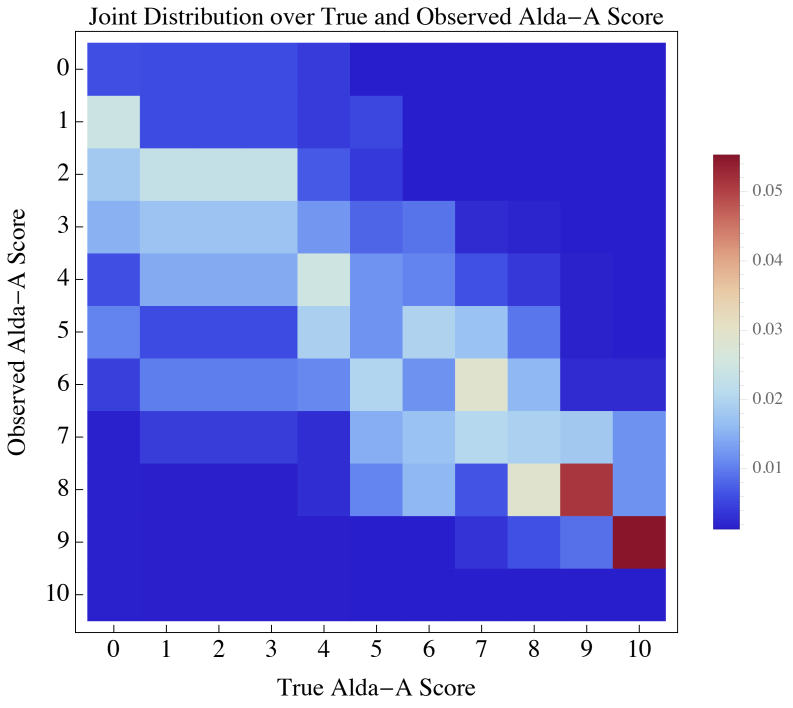

In[47]:=

```
Pxy[α_]:= (Q[α]×Table[1/11, {i, 11}, {i, 11}]);
Px[α_]:= (Total@Pxy[α]);
Py[α_]:= Total[Pxy[α]ᵀ];
Jxy[α_]:= J[α];
Jx[α_]:= (Total@Jxy[α]);
Jy[α_]:= Total[Jxy[α]ᵀ];
```

In[53]:=

```
AldaMI[α_]:=N@Total[Total[Pxy[α] Log[Pxy[α]/(Px[α]⊗Py[α])]]]
DiscreteAldaMI[α_]:=N@Total[Total[Jxy[α] Log[Jxy[α]/(Jx[α]⊗Jy[α])]]]
```

In[55]:=

```
miaaldafig = Plot[
    {AldaMI[α], DiscreteAldaMI[α]}, {α, 2, 10},
    PlotStyle→63, Frame→True, FrameStyle→Directive[Black,15],
    FrameLabel→{"α", "Mutual Information"},
    PlotTheme→"Scientific",
    PlotLegends→Placed[{
        Style["Raw", Black, 16],
        Style["Dichotomized", Black, 16]},
        {0.75, 0.75}],
    ImageSize→400];
(*Export["~/Desktop/mutualinformationaldascoresA.png",
        miaaldafig,
        ImageResolution→500,
        "AllowRasterization"→True];*)

partA = GraphicsGrid[{{DiscretePlot3D[Pxy[0.0001][[i,j]],{i,1,11}, {j,1,11}, ExtentSize→1
 PlotLabel→Style["Continuous Scale (α = 0)\nI_α[x_o||x_*]="<>ToString[Round[Re@AldaMI[0.0001]
 PlotTheme→"Scientific", ColorFunction→"ThermometerColors", AxesLabel→{"x_*", "x_o", "p(x_o,x
 AxesStyle→Directive[Black, 13]],
 DiscretePlot3D[Jxy[11/2×0.0001][[i,j]],{i,1,2}, {j,1,2}, ExtentSize→1/2,
 PlotLabel→Style["Discretized Scale (α = 0)\nI_γ[y_o||y_*]="<>ToString[Round[DiscreteAldaMI[0
 PlotTheme→"Scientific", ColorFunction→"ThermometerColors", AxesLabel→{"y_*", "y_o", "p(y_o,y
 AxesStyle→Directive[Black, 13],
 Ticks→{{{1, 2}, {"Li^(-)", "Li^(+)"}}^T, {{1, 2}, {"Li^(-)", "Li^(+)"}}^T, Automatic}]}}, ImageSize

partB = GraphicsGrid[{{DiscretePlot3D[Pxy[10][[i,j]],{i,1,11}, {j,1,11}, ExtentSize→1/2,
 PlotLabel→Style["Continuous Scale (α = 10)\nI_α[x_o||x_*]="<>ToString[Round[Re@AldaMI[10], 0
 PlotTheme→"Scientific", ColorFunction→"ThermometerColors", AxesLabel→{"x_*", "x_o", "p(x_o,x
 AxesStyle→Directive[Black, 13]],
 DiscretePlot3D[Jxy[11/2×10][[i,j]],{i,1,2}, {j,1,2}, ExtentSize→1/2,
 PlotLabel→Style["Discretized Scale (α = 10)\nI_γ[y_o||y_*]="<>ToString[Round[DiscreteAldaMI[
 PlotTheme→"Scientific", ColorFunction→"ThermometerColors", AxesLabel→{"y_*", "y_o", "p(y_o,y
 AxesStyle→Directive[Black, 13],
 Ticks→{{{1, 2}, {"Li^(-)", "Li^(+)"}}^T, {{1, 2}, {"Li^(-)", "Li^(+)"}}^T, Automatic}]}}, ImageSize

partC = GraphicsGrid[{{DiscretePlot3D[Pxy[100][[i,j]],{i,1,11}, {j,1,11}, ExtentSize→1/2,
 PlotLabel→Style["Continuous Scale (α = 100)\nI_α[x_o||x_*]="<>ToString[Round[Re@AldaMI[100],
 PlotTheme→"Scientific", ColorFunction→"ThermometerColors", AxesLabel→{"x_*", "x_o", "p(x_o,x
```

```
 AxesStyle→Directive[Black, 13]],
 DiscretePlot3D[Jxy[ 11/2 ×100][[i,j]],{i,1,2}, {j,1,2}, ExtentSize→1/2,
 PlotLabel→Style["Discretized Scale (α = 100)\n𝕀γ[yo||y*]="<>ToString[Round[DiscreteAldaMI
 PlotTheme→"Scientific", ColorFunction→"ThermometerColors", AxesLabel→{"y*", "yo", "p(yo,y
 AxesStyle→Directive[Black, 13]],
 Ticks→{{{1, 2}, {"Li(-)", "Li(+)"}}ᵀ, {{1, 2}, {"Li(-)", "Li(+)"}}ᵀ, Automatic}]]}}, ImageSize

gridlab[x_] := Style[x, Black, Bold, 28, FontFamily→"Times New Roman"]
(*ascoreasymmetrygrid = Grid[{
    gridlab[#]&/@{"A", "B"},
    {partA, partB},
    gridlab[#]&/@{"C", "D"},
    {partC, miaaldafig}}];
Export["ascoreasymmetrygrid.pdf", ascoreasymmetrygrid, "AllowRasterization"→True];*)

partA
partB
partC
miaaldafig
```

Out[60]=

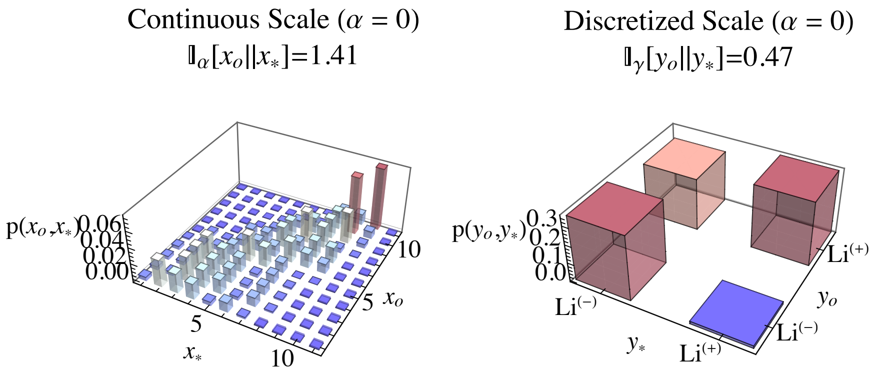

Out[61]=

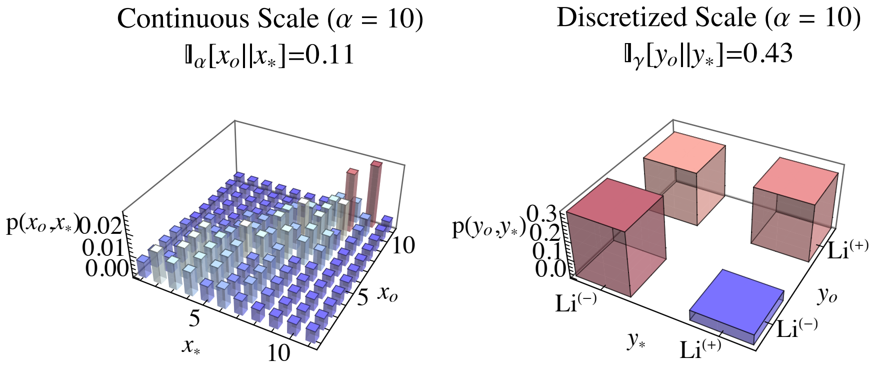

Out[62]=

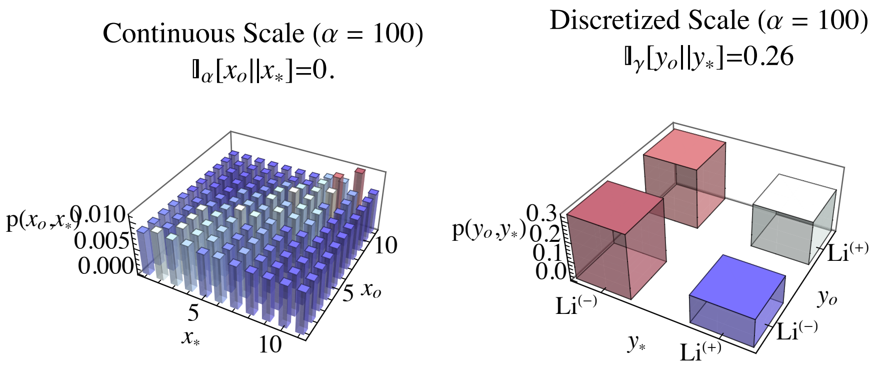
Continuous Scale (α = 100)
𝕀α[xo||x*]=0.
p(xo,x*)
0.010
0.005
0.000
5
x*
10
10
5 xo
Discretized Scale (α = 100)
𝕀γ[yo||y*]=0.26
p(yo,y*)
0.3
0.2
0.1
0.0
Li(−)
y*
Li(+)
Li(+)
yo
Li(−)

Out[63]=

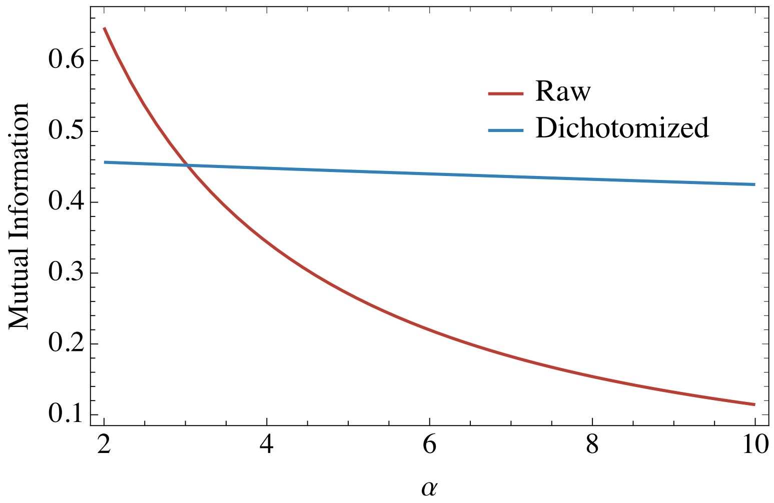
Mutual Information
0.6
0.5
0.4
0.3
0.2
0.1
2
4
6
8
10
α
Raw
Dichotomized
