# Supplementary material for: Asymmetrical reliability of the Alda score favours a dichotomous representation of lithium responsiveness
Source: PLoS One. 2020 Jan 27;15(1):e0225353. doi: 10.1371/journal.pone.0225353 (PMC6984707; doi:10.1371/journal.pone.0225353)
Supplement: S7 File — PDF version of S4 File for those without Mathematica license. (PDF) [file pone.0225353.s009.pdf]

Code for Simulations in *Asymmetrical Reliability of the Alda Score favours a Dichotomous Representation of Lithium Response*

Abraham Nunes (nunes@dal.ca). *Dalhousie University, Halifax, Nova Scotia, Canada*

Preliminaries

In[31]:=
```
SeedRandom[865];
```

Data generating process

The reflection function simply keeps points within a certain bounded box [l, u] by reflecting points that exceed those bounds back into the box.

In[2]:=
```
Reflection[l_, u_][x_]:=Max[{Min[{x, Max[{2u-x, l}]}], Min[{2l-x, u}]}]
```

Now we define the data generators. **Note that in this document code, the "*β*" variable does not directly correspond to the one denoted in the main paper.** The *β* in the main paper was defined as such simply for notational convenience.

First, we have the function that generates the asymmetrically variable diagonal.

In[3]:=
```
Data[n_:100, β_:0.75][σ_] := Table[
    {x, Reflection[0, 10][x+ 1/(1 + ⅇ^(-β x+5)) RandomVariate[UniformDistribution[{-σ ,σ }]]]},
    {x, 0, 10, 10/(n-1)}];
```

Second, we have the function that generates the symmetrically variable diagonal.

In[4]:=
```
SymmetricData[n_:100] := Function[σ,
    d = Table[{x, x+RandomVariate[UniformDistribution[{-σ ,σ }]]}, {x, 0, 10, 10/(n-1)}];
    d[[All, 2]] = 10 (d[[All, 2]]-Min[d[[All, 2]]])/(Max[d[[All, 2]]-Min[d[[All, 2]]]]);
    d
];
```

Now, we define the function that combines the diagonal with some global noise uniformly spread across the [0,10] interval. First, we specify this for the asymmetrically reliable synthetic data.

In[5]:=
```
NoisyData[n_:2, β_:1, ω_:1]:=Function[σ,
    Module[{data, noise},
        data = Data[Round[(1-ω) n] ,β][σ];
        noise = RandomReal[{0, 10}, {Round[ω n], 2}];
        Catenate[{data, noise}]
]];
```

Second, we specify this for the symmetrically reliable synthetic data.

In[6]:=
```
SymmetricNoisyData[n_:2, ω_:1]:=Function[σ,
    Module[{data, noise},
        data = SymmetricData[Round[(1-ω) n]][σ];
        noise = RandomReal[{0, 10}, {Round[ω n], 2}];
        Catenate[{data, noise}]
]]
```

Data Demonstration Figure

Now, we demonstrate the synthetic data graphically. The “iconized” portions of the code below are merely style elements for the plot that are important for neither conceptual interpretation nor reproducibility.

In[7]:=
```
asymmetricdatademofig = Show[GraphicsGrid[Table[Show[
    ListPlot[NoisyData[500, 0.75, ω][σ], ... ], Graphics[...] ],
{ω, {0.1, 0.3, 0.5}},{σ, {0.5, 5, 10, 20}}], ... ], Rule[...] ];
```

In[8]:=
```
symmetricdatademofig = Show[GraphicsGrid[Table[Show[
    ListPlot[SymmetricNoisyData[500, ω][σ], ... ], Graphics[...] ],
{ω, {0.1, 0.3, 0.5}},{σ, {0.5, 5, 10, 20}}], ... ],
Rule[...] ];
```

In[9]:=
```
datademofig = Grid[{{asymmetricdatademofig}, {symmetricdatademofig}}]
```

Out[9]=

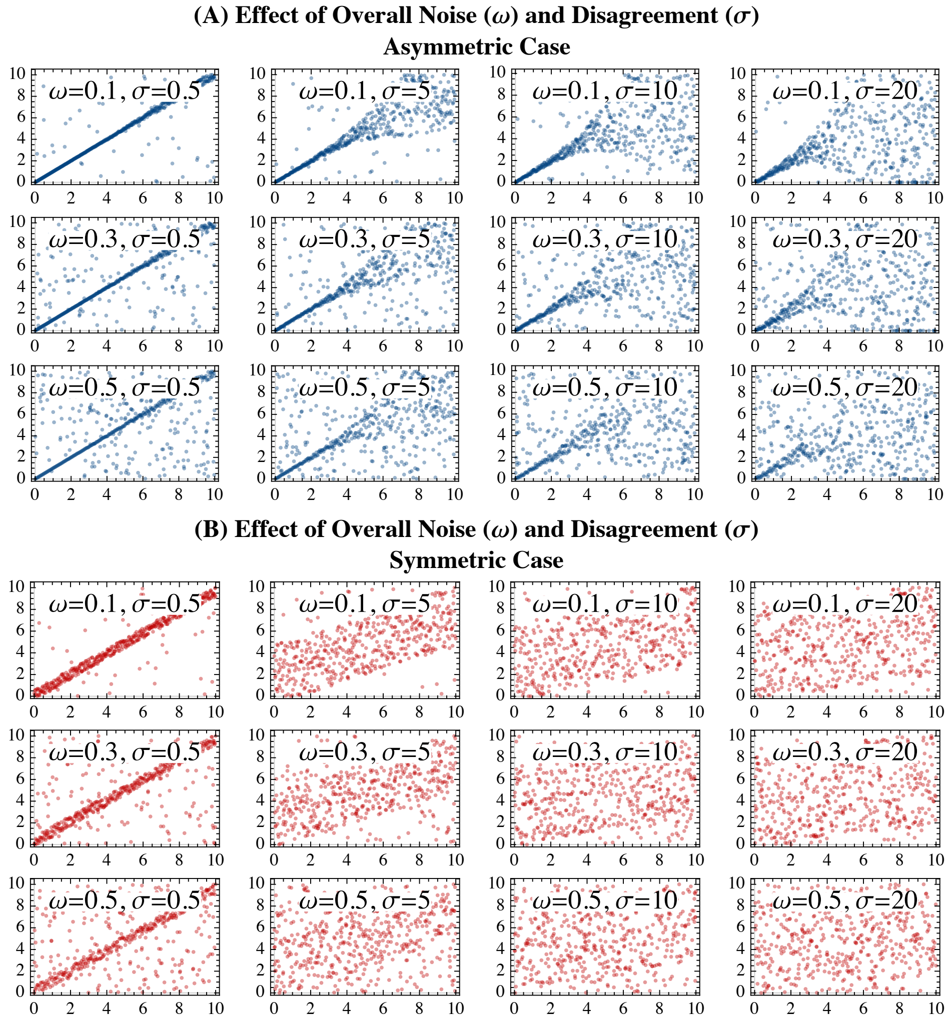


```
Export["~/Desktop/datademo.png", datademofig,
  ImageResolution → 350, "AllowRasterization" → True];
```

Computing Mutual Information

Functions to compute continuous mutual information

First, we define the kernel density estimation function.

In[10]:=
```
kde[𝒟_] := SmoothKernelDistribution[𝒟, "Scott"];
```

Second, we define a function to numerically integrate the learned kernel distribution.

In[11]:=
```
ContinuousMI[p_][l_:0, u_:10]:= NIntegrate[
    PDF[p, {x, y}]Log[PDF[p, {x, y}] / (PDF[MarginalDistribution[p, 1],x]×PDF[MarginalDistribution[p, 2], y
{x,l, u},{y, l, u}, Method→"AdaptiveMonteCarlo"]
```

Functions to compute discrete mutual information

First, we discretize the data.

In[12]:=
```
DiscretizeData[𝒟_][τ_]:=Table[{ 1   𝒟[[i,j]]<τ
                                  0   True      ,{i, Length@𝒟}, {j, 2}]
```

Second, we compute a joint distribution over the discretized classes.

In[13]:=
```
DiscreteDist[𝒟_]:=Function[τ,
     Module[{tally, Txy},
         tally = Tally[DiscretizeData[𝒟][τ]];
         Txy = (Select[tally, #[[1]] =={1,0}&][[1,2]]  Select[tally, #[[1]] =={0,0}&][[1,2]]
                Select[tally, #[[1]] =={1,1}&][[1,2]]  Select[tally, #[[1]] =={0,1}&][[1,2]]
         Txy / Total[Total[Txy]]
 ]];
```

Finally, we compute the mutual information of the discrete distribution.

In[14]:=
```
DiscreteDistMI[𝒟_] := Function[τ,
    Module[{Jxy , Jx, Jy},
        Jxy = DiscreteDist[𝒟][τ];
        Jx = Total[Jxy];
        Jy = Total[Jxy^T];
        N@(Sum_{i=1}^{2} Sum_{j=1}^{2} Jxy[[i, j]] Log[Jxy[[i, j]] / (Jy[[i]] × Jx[[j]])])
    ]];
```

Evaluation

Statistical helper functions

Just a function to compute the confidence intervals of a set of sequences.

In[15]:=
```
ConfidenceInterval[α_:0.05, tails_:2]:=Function[X,
    Module[{m, confr},
        m = Mean[X];
        confr = Abs[InverseSurvivalFunction[NormalDistribution[],1-α/tails]](StandardDeviat
        {m-confr, m, m+confr}
    ]]
```

Experimental parameters

In[16]:=
```
sigmas = Table[σ, {σ, {0.5,5,10, 20}}];
omegas = Table[ω, {ω, {0.1,0.3,0.5}}];
thresholds = Range[1,9];
```

Comparison of the discrete and continuous mutual information for **Asymmetrically** reliable data

Continuous mutual information:

In[19]:=
```
contmi = Table[ContinuousMI[kde[NoisyData[750, 0.75, ω][σ]]][], {ω, omegas},{σ, sigmas}];
```

Discrete mutual information:

In[20]:=
```
discretemi = Table[
    {τ, DiscreteDistMI[NoisyData[750, 0.75, ω][σ]][τ]},
    {i,1,10},{ω,omegas},{σ,sigmas},{τ,thresholds}];
discretemi = Table[
    Select[discretemi[[i, j, k]], #[[2]] ≠ "Indeterminate" &],
    {i,Length@discretemi}, {j,Dimensions[discretemi][[2]]}, {k,Dimensions[discretemi][[3]
```

Results Figure

In[22]:=
```
mivsnoisefig = Show[GraphicsGrid[ArrayReshape[{Table[Show[
Table[ListLinePlot[ConfidenceInterval[][discretemi[[All, i, j, All]]],
    PlotLabel→Style["σ="<>ToString[sigmas[[j]]], Black,14, FontFamily→"Times"],
    PlotStyle→{Opacity[0], ColorData[63][i]}, Filling→ {1→{3}},
    FillingStyle→Opacity[0.2,ColorData[63][i]],
    GridLines→{{Mean[Range[0, 10]]}, None},
    PlotTheme→"Scientific", AspectRatio→1], {i,3}],
Table[Plot[contmi[[i,j]], {τ, 0, 10}, PlotStyle→Directive[Dashed, ColorData[63][i]]], {i,
 Frame→True, FrameStyle→Directive[Black, 14],FrameLabel→{"Threshold (τ)", "MI"}],
  {j, 4}]}, {1, 4}], Spacings→0],
PlotLabel→Style["(A) Asymmetric Agreement Noise", Black, Bold, 16, FontFamily→"Times"]]
```

Out[22]=

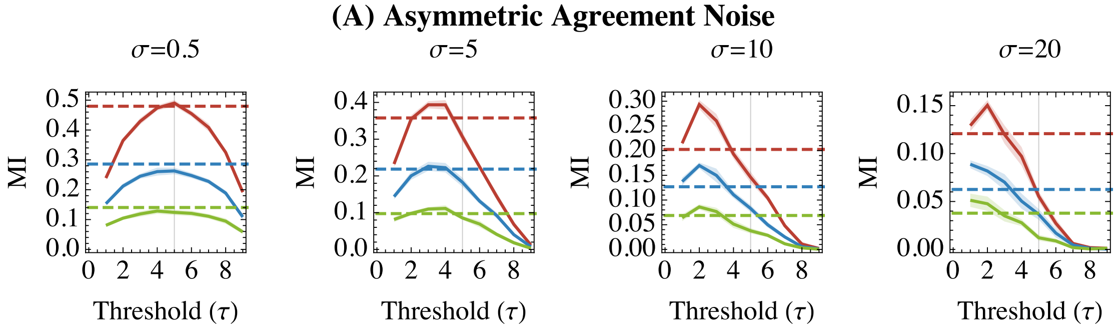

Comparison of the discrete and continuous mutual information for **Symmetrically** reliable data

Continuous mutual information:

In[23]:=
```
symcontmi = Table[ContinuousMI[kde[SymmetricNoisyData[750, ω][σ]]][], {ω, omegas},{σ,sigma
```

Discrete mutual information

In[24]:=
```
symdiscretemi = Table[
    {τ, DiscreteDistMI[SymmetricNoisyData[750, ω][σ]][τ]},
    {i,1,10},{ω,omegas},{σ,sigmas},{τ,thresholds}];
symdiscretemi = Table[
    Select[symdiscretemi[[i, j, k]], #[[2]] ≠ "Indeterminate" &],
    {i,Length@symdiscretemi}, {j,Dimensions[symdiscretemi][[2]]}, {k,Dimensions[symdiscre
```

Results figure for symmetrical experiment

In[26]:=

```
symmetricmivsnoisefig = Show[GraphicsGrid[ArrayReshape[{Table[Show[
Table[ListLinePlot[ConfidenceInterval[][symdiscretemi[[All, i, j, All]]],
    PlotLabel→Style["σ="<>ToString[sigmas[[j]]], Black,14, FontFamily→"Times"],
    PlotStyle→{Opacity[0], ColorData[63][i]}, Filling→ {1→{3}},
    FillingStyle→Opacity[0.2,ColorData[63][i]],
    GridLines→{{Mean[Range[0, 10]]}, None},
    PlotTheme→"Scientific", AspectRatio→1], {i,3}],
 Table[Plot[symcontmi[[i,j]], {τ, 0, 10}, PlotStyle→Directive[Dashed, ColorData[63][i]]],
 Frame→True, FrameStyle→Directive[Black, 14],FrameLabel→{"Threshold (τ)", "MI"}],
  {j, 4}]}, {1, 4}],
Spacings→0],
PlotLabel→Style["(B) Symmetric Agreement Noise", Black, Bold, 16, FontFamily→"Times"]]
```

Out[26]=

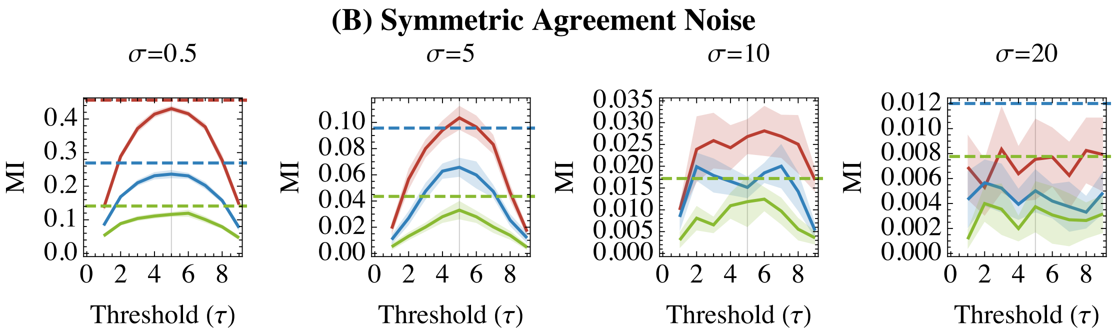

Composite figure for results of the symmetrical and asymmetrical data experiments

In[27]:=
```
syntheticresultsfig = Legended[
   Grid[{{mivsnoisefig}, {symmetricmivsnoisefig}}],
   LineLegend[63, Table["ω="<>ToString[Round[ω, 0.01]], {ω, {0.1, 0.3, 0.5}}]]]
```

Out[27]=

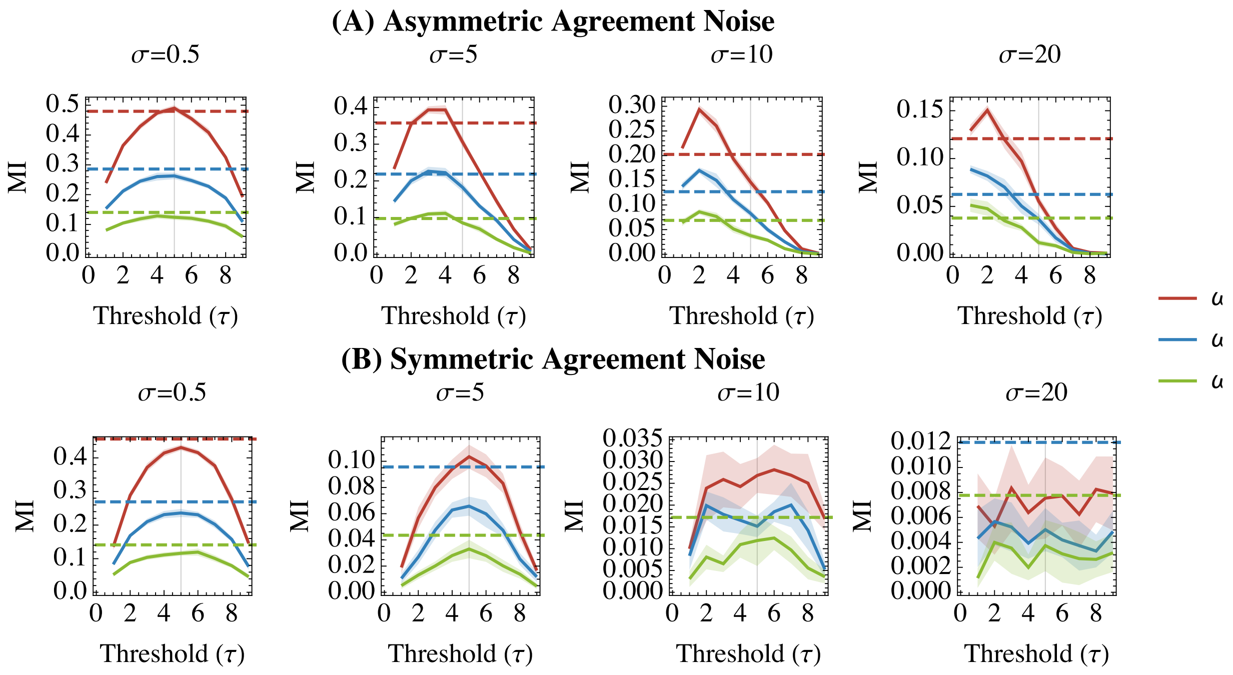

In[∘]:=
```
Export["~/Desktop/mivsnoisefig.png", syntheticresultsfig,
  ImageResolution → 350, "AllowRasterization" → True]
```

Out[∘]= ~/Desktop/mivsnoisefig.png

Sanity Check on the Regular Grid

Generate the grid.

In[28]:=
```
𝒟reg = Flatten[Table[{i,j}, {i, 0, 10, 0.01}, {j, 0, 10, 0.01}],1];
```

Compute the continuous mutual information (should be close to or equal to zero):

In[29]:=
```
ContinuousMI[kde[𝒟reg]][]
```

Out[29]= -9.98621×10^{-17}

Now compute the discrete mutual information (it should be very close or equal to zero across the whole range):

In[30]:=
```
dmi = Table[DiscreteDistMI[𝒟reg][τ], {τ, thresholds}]
```

Out[30]=
```
{0., 0., 0., 0., 0., 0., 0., 0., 0.}
```

Printed by Wolfram Mathematica Student Edition
